# The Central Clock Neurons Regulate Lipid Storage in *Drosophila*

**DOI:** 10.1371/journal.pone.0019921

**Published:** 2011-05-18

**Authors:** Justin R. DiAngelo, Renske Erion, Amanda Crocker, Amita Sehgal

**Affiliations:** 1 Department of Neuroscience, The University of Pennsylvania, Philadelphia, Pennsylvania, United States of America; 2 Howard Hughes Medical Institute, Hofstra University, Hempstead, New York, United States of America; 3 Department of Biology, Hofstra University, Hempstead, New York, United States of America; University of Houston, United States of America

## Abstract

A proper balance of lipid breakdown and synthesis is essential for achieving energy homeostasis as alterations in either of these processes can lead to pathological states such as obesity. The regulation of lipid metabolism is quite complex with multiple signals integrated to control overall triglyceride levels in metabolic tissues. Based upon studies demonstrating effects of the circadian clock on metabolism, we sought to determine if the central clock cells in the *Drosophila* brain contribute to lipid levels in the fat body, the main nutrient storage organ of the fly. Here, we show that altering the function of the *Drosophila* central clock neurons leads to an increase in fat body triglycerides. We also show that although triglyceride levels are not affected by age, they are increased by expression of the amyloid-beta protein in central clock neurons. The effect on lipid storage seems to be independent of circadian clock output as changes in triglycerides are not always observed in genetic manipulations that result in altered locomotor rhythms. These data demonstrate that the activity of the central clock neurons is necessary for proper lipid storage.

## Introduction

Throughout evolution, the ability of humans to convert glucose to triglyceride for long-term storage has provided a competitive advantage during times of famine. However, in our current Western society where food is abundantly available, this thrifty phenotype has resulted in excess fat accumulation leading to 65% of adults in the United States being overweight and 30% being obese [1]. Clearly, a proper balance of the synthesis and breakdown of lipids is essential for reaching metabolic homeostasis, but the mechanisms responsible for controlling these processes are still not fully understood.

The regulation of lipid metabolism is a very complex process, utilizing a number of signals and pathways leading to lipid synthesis, breakdown or both [2]. Recent research has focused on understanding the regulation of lipid metabolism in liver and adipose tissue by the brain (reviewed in [3], [4]). In mammals, the arcuate nucleus (ARC) of the hypothalamus serves as a main regulator of energy homeostasis by integrating signals from many circulating hormones. The ARC also receives neural inputs from other regions of the hypothalamus, one of these being the suprachiasmatic nucleus (SCN), the site of the central circadian clock [5]. The circadian system is, in fact, known to be a major regulator of metabolic activity, with profound metabolic phenotypes reported in clock mutant animals [6], [7]. However, analysis of underlying mechanisms has focused on autonomous effects of clocks located in metabolic tissues such as the control of gene expression by such clocks as well as interactions between clock proteins and metabolic factors in these tissues [8], [9], [10]. Despite the connection between the ARC and the SCN, little is known about the contribution of the central clock to metabolic processes.

The fruit fly, *Drosophila melanogaster*, is a well-established model of circadian rhythms and has recently become a powerful model to study the regulation of metabolism [11]. In *Drosophila*, as in mammals, the central clock is found in specific neurons of the brain, but clocks also exist in other body tissues [12], [13], [14]. However, effects of these different clocks on metabolic activity are poorly understood. We showed recently that the *Drosophila* fat body (equivalent of mammalian liver and adipose tissue) contains a circadian clock, which regulates the storage of glycogen and triglycerides [15]. Clocks in neurons also affect glycogen storage, but the specific neurons responsible were not identified and the control of triglyceride levels by neuronal clocks was not assessed [15]. Here, we sought to explore a role of the central clock neurons in the accumulation of lipids. We report that knocking down the function of the circadian gene, *Clock (Clk*) in central clock cells leads to increased triglycerides in the fly's fat body. We observe a similar phenotype when we trigger premature degeneration in these neurons. However, triglyceride levels are normal in arrhythmic flies that express the heat-sensitive ion channel dTRPA1 in the PDF neurons and in *Pdf^01^* mutants, suggesting that these neurons control fat storage independently of the circadian rest∶activity output. In addition, over-expression of the clock gene, *timeless (tim)*, in these neurons does not affect triglycerides although it reduces behavioral rhythmicity. Together, these findings indicate a non-circadian role for the central clock neurons in controlling overall energy homeostasis.

## Materials and Methods

### Fly genetics

Flies were grown on standard cornmeal molasses medium at room temperature. Prior to each experiment, 0–3 day old females were entrained for 2–3 days in a 12 h∶12 h light∶dark cycle at 25°C. For dTRPA1 experiments, flies were reared at 18–21°C and 0–3 day old flies were shifted to 27°C in 12 h∶12 h light∶dark conditions for seven days before being assayed. Fly strains used in this study include: *iso^31^* (Bloomington #5905), *Clk^Jrk^* [16], *cyc^01^* [17], *tim^01^* [18], *per^0^* [19], *Pdf-Gal4* [20], *UAS-ClkΔ* [14], *UAS-Clk*RNAi (VDRC #42834), *UAS-Aβ42ArcM* [21], *UAS*-*tim* [22], *UAS*-dTRPA1 [23] and *Pdf^01^* [20].

### Triglyceride and protein measurements

Fat bodies were dissected from abdomens of 4–7d old mated females as described previously [24]. Fat bodies were homogenized in lysis buffer containing 140 mM NaCl, 50 mM Tris-HCl, pH 7.4, 0.1% Triton X and 1X protease inhibitor cocktail (Roche Diagnostics, Mannheim, Germany) and triglyceride and protein measurements were made using the triglyceride LiquiColor kit (Stanbio Laboratory, Boerne, TX) and bicinchoninic acid protein assay kit (Thermo Scientific, Waltham, MA), respectively, according to manufacturer's instructions. Single balancer chromosomes were used to identify Gal4 and transgene only control flies for these experiments; since the single balancer chromosomes had no difference in triglycerides compared to wildtype chromosomes (data not shown), both wildtype and balancer chromosomes were denoted as “+” for clarity. Samples were collected at Zeitgeber time (ZT) 0, 4, 8, 12, 16, and 20 and averaged across all timepoints unless otherwise noted.

### Behavior measurements

Flies were entrained in 12 h∶12 h light∶dark conditions for at least two days before assessing circadian behavior using the previously described DAMS system (Trikinetics, Waltham MA). The flies were maintained on 5% sucrose and 2% agar throughout the recording. Activity was collected in five-minute bins for a minimum of five days in constant darkness and analyzed using Clocklab software (Actimetrics) as described previously [22].

### Feeding measurements

Food consumption was measured over a 24-hour period using a modified version of the CAFÉ assay as described [15]. Briefly, flies were entrained in 12 h∶12 h light∶dark conditions for at least two days on regular food at 25°C. On the day of the assay, flies were flipped into vials with 1% agar as a water source and 5% sucrose in 5 µl calibrated glass micropipettes (VWR, West Chester, PA) as their only food source. After 24 hours, the amount of liquid food consumed was measured and was adjusted to take into account evaporation, which was measured using a sucrose-filled micropipette in a 1% agar vial without flies.

## Results

### Fat body triglycerides are unchanged across the circadian day and in clock mutants

**Figure 1:**
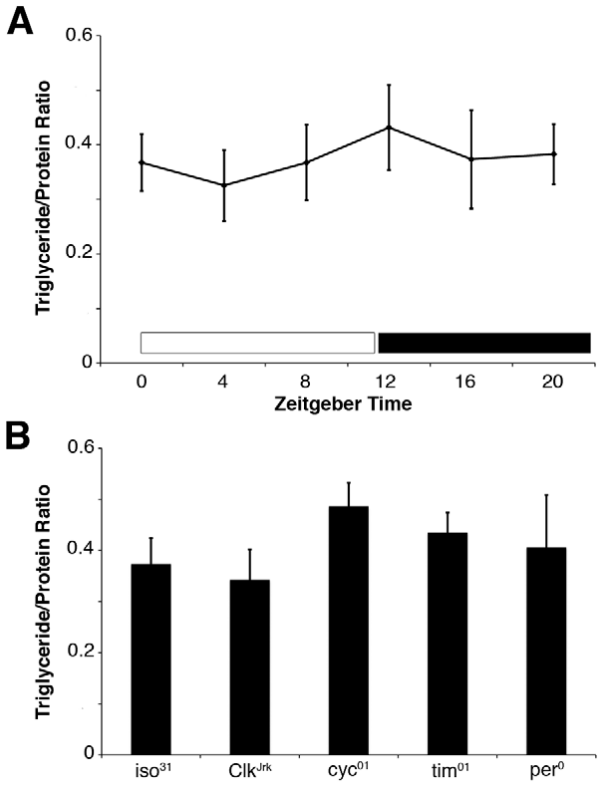
Fat body triglycerides fail to cycle and are unaffected in circadian clock mutants. (**A**) Triglyceride/protein ratios of fat bodies dissected from 4–7 day old female *iso^31^* (wildtype) flies at Zeitgeber time (ZT) 0, 4, 8, 12, 16, and 20. (**B**) Triglyceride/protein ratios of fat bodies dissected from 4–7 day old *Clk^Jrk^*, *cyc^01^*, *tim^01^*, and *per^0^* females and compared to *iso^31^* controls. Each experiment was performed at least three times with greater than 65 animals assayed in total, and values represent mean ± SEM.

To investigate the role of the circadian system in regulating lipid accumulation in *Drosophila*, we started by measuring triglycerides at different times of day as many circadian-regulated processes proceed in a cyclic manner. In *Drosophila*, the majority of stored triglycerides are found in the fat body, an organ analogous to the mammalian liver and adipose tissue [25]. Thus, we measured triglyceride levels in abdominal fat bodies dissected from wildtype animals entrained to a 12 h∶12 h light∶dark cycle. In contrast to feeding that occurs rhythmically over the course of the day [15], fat body triglycerides remained constant at all time points tested (Fig. 1A).

Although fat body triglycerides fail to cycle, it is possible that the circadian clock controls overall lipid levels. To address this question, we measured triglycerides in fat bodies of clock mutant animals, *Clk^Jrk^*, *cyc^01^*, *tim^01^* and *per^0^*, and compared these to an isogenized *w^1118^* wildtype control strain (*iso^31^*), a background into which all of the clock mutant strains were outcrossed at least 5 times. Triglycerides were similar in all of these animals suggesting that an absence of circadian clocks in all cells does not perturb fat body lipid levels (Fig. 1B).

### Inhibiting the activity of the *Clk* transcription factor in PDF neurons increases triglycerides

**Figure 2:**
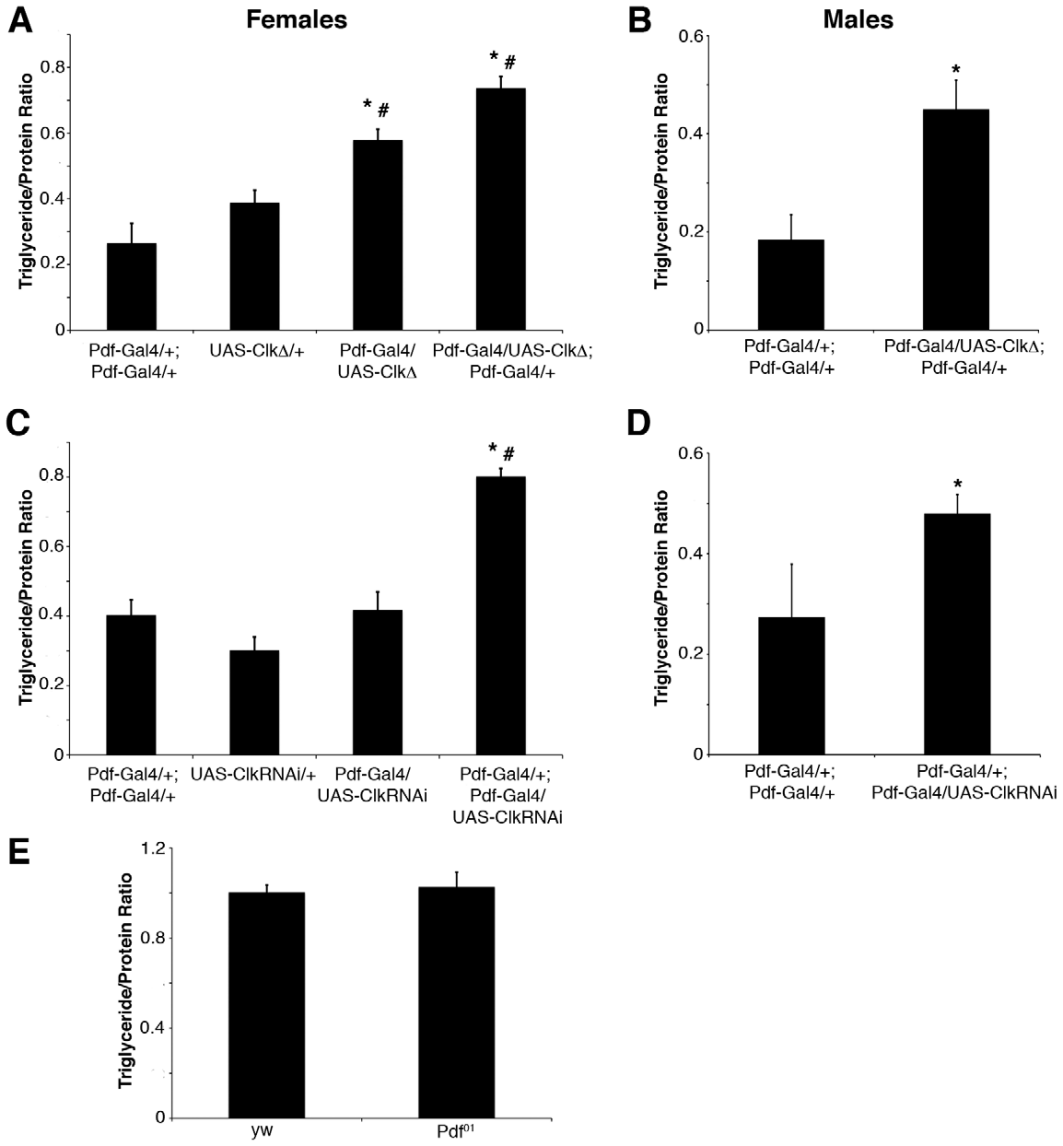
Inhibiting *Clk* gene function in the PDF neurons results in increased fat body triglycerides. (A) Triglyceride/protein ratios of fat bodies dissected from 4–7 day old *Pdf-Gal4/UAS-ClkΔ* and *Pdf-Gal4/UAS-ClkΔ*; *Pdf-Gal4/+* female flies and compared to *Pdf-Gal4/+*; *Pdf-Gal4/+* and *UAS-ClkΔ/+* controls. (B) Triglyceride/protein ratios of fat bodies dissected from 4–7 day old *Pdf-Gal4/UAS-ClkΔ*; *Pdf-Gal4/+* male flies and compared to *Pdf-Gal4/+*; *Pdf-Gal4/+* controls. (C) Triglyceride/protein ratios of fat bodies dissected from 4–7 day old *Pdf-Gal4/+*; *UAS-Clk*RNAi*/+* and *Pdf-Gal4/+*; *Pdf-Gal4/UAS-Clk*RNAi female flies and compared to *Pdf-Gal4/+*; *Pdf-Gal4/+* and *UAS-Clk*RNAi*/+*controls. (D) Triglyceride/protein ratios of fat bodies dissected from 4–7 day old *Pdf-Gal4/+*; *Pdf-Gal4/UAS-Clk*RNAi male flies and compared to *Pdf-Gal4/+*; *Pdf-Gal4/+* controls. (E) Triglyceride/protein ratios of fat bodies dissected from 4–7 day old *y w*; *Pdf^01^* female flies and compared to *y w* controls. (A,C,E) Each experiment was performed at least three times with greater than 100 animals in total and values represent mean ± SEM. *, # p<0.05 assessed by one-way ANOVA followed by post-hoc Tukey test compared to *Pdf-Gal4* only and transgene only genotypes, respectively. (B,D) Each experiment was performed three times with greater than 40 animals in total, and the animals were dissected between ZT6-9. A representative experiment is shown and values represent mean ± SD. * p<0.05 assessed by Student's t-test.

We recently demonstrated that a peripheral clock in metabolic tissues promotes glycogen accumulation, while neuronal clocks oppose this action. Because of these opposing effects, circadian mutants that lack clocks in both metabolic and neuronal tissues have normal glycogen levels [15]. A similar regulation of lipid levels is suggested by the finding that disruption of the clock in metabolic tissues decreases triglycerides [15] while clock mutants have normal levels (Fig. 1B). To determine if the central clock in particular affects fat body triglycerides, we used the Gal4/UAS system to specifically inhibit this clock. The central clock in flies is located in the ventral lateral neurons (LNvs), which specifically express the neuropeptide, pigment dispersing factor (PDF). As previously reported, expression of a dominant-negative form of the circadian transcription factor Clock (CLKΔ) in these neurons using *Pdf-Gal4* inhibits the central clock [14]. Interestingly, ectopic expression of CLKΔ in LNvs led to an increase in fat body triglycerides, in particular when two copies of *Pdf-Gal4* were used to express CLKΔ (Fig. 2A, 2B). A similar phenotype was observed when a *Clk*RNAi construct was expressed in these neurons using two copies of *Pdf-Gal4* (Fig. 2C, 2D). While the expression of CLKΔ eliminates overt rhythms of rest∶activity, the RNAi construct resulted only in a lengthening of circadian period, indicating that it preserves general clock function (Table 1, [14]). Nevertheless, there was a significant effect on lipid levels.

**Table 1:** Activity rhythms in flies expressing ClkRNAi, Aβ42ArcM, tim, and dTRPA1 in PDF neurons.

Genotype	Number tested (% arrhythmic)	Period Length (hrs ± SD)	FFT (± SD)
*Pdf-Gal4/+; Pdf-Gal4/+*	130 (18.5)	23.8±0.4	0.093±0.1
*UAS-Clk*RNAi*/+*	45 (15.6)	24.0±0.6	0.069±0.04
*UAS-*Aβ42ArcM*/+*	48 (8.3)	24.2±0.4	0.065±0.03
*Pdf-Gal4/+; Pdf-Gal4>Clk*RNAi	44 (4.5)	24.9±0.4*	0.081±0.04
*Pdf-Gal4/+; Pdf-Gal4>*Aβ42ArcM	44 (18)	24.2±0.5	0.058±0.03
*Pdf-Gal4>tim; Pdf-Gal4/+*	47 (36.2)	24.3±0.6	0.055±0.04
# *Pdf-Gal4/+; Pdf-Gal4/+*	48 (27.1)	24.0±0.3	0.102±0.04
# *UAS*-dTRPA1*/+*	47 (12.8)	23.6±0.3	0.126±0.06
# *Pdf-Gal4*>dTRPA1; *Pdf-Gal4/+*	43 (95.3)	24.7±1.8	0.054±0.02

Activity rhythm data of 1–2 week old female flies.

# Data were collected from flies that were housed at 27°C throughout the entire experiment.

*p<0.05 assessed by one-way ANOVA followed by post-hoc Tukey test compared to *Pdf-Gal4* only and *UAS-Clk*RNAi genotypes.

The neuropeptide PDF is not only a marker for the LNvs, it is also an important output of these neurons required for the maintenance of rest∶activity rhythms [20]. PDF is positively regulated by the circadian transcription factors *Clk* and *cyc* [26]; therefore, it is a good candidate for mediating the effects of CLK on triglycerides. However, triglyceride levels were normal in *Pdf^01^* mutants (Fig. 2E), suggesting that other outputs are responsible for this effect. Together these data indicate that the central clock neurons affect lipid accumulation in the fat body, but do not do so through PDF.

### Expression of a neurodegeneration-inducing protein in clock neurons results in elevated triglycerides

**Figure 3:**
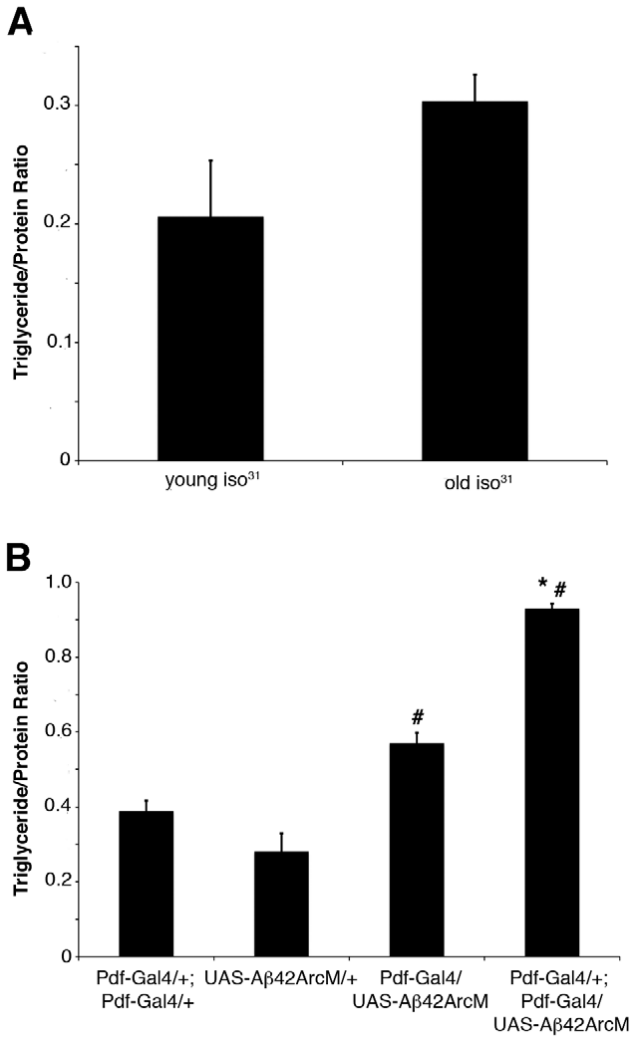
The effects of age and neurodegeneration on fat body triglycerides. (A) Triglyceride/protein ratios of fat bodies dissected from 50–55 day old wild type females (old *iso^31^*) at ZT8 and compared to 4–7 day old wild type females (young *iso^31^*) at ZT8. (B) Triglyceride/protein ratios of fat bodies dissected from 4–7 day old *Pdf-Gal4/+*; *UAS-*Aβ42ArcM*/+* and *Pdf-Gal4/+*; *Pdf-Gal4/UAS-*Aβ42ArcM female flies and compared to *Pdf-Gal4/+*; *Pdf-Gal4/+* and *UAS-*Aβ42ArcM*/+*controls. Each experiment was performed at least three times and values represent mean ± SEM. The data in (A) were obtained from 36 animals and the data in (**B**) from greater than 100 animals. *, # p<0.05 assessed by one-way ANOVA followed by post-hoc Tukey test compared to *Pdf-Gal4* only and transgene only genotypes, respectively.

A physiological context during which normal circadian rhythms begin to break down is during aging. Specifically in *Drosophila*, the very robust locomotor activity rhythms decline as flies age [27]. Given that metabolic changes also occur with age, it is possible that triglyceride levels are affected by the aging process. To test this hypothesis, we aged wildtype *iso^31^* flies 50–55 days and compared their fat body triglycerides to those of young one-week-old flies. The triglyceride levels of the aged flies trended to be higher than the young ones, although the difference did not reach statistical significance (Fig. 3A).

Our data indicate that normal aging does not have a profound effect on lipid accumulation. However, a number of age-related syndromes, such as neurodegenerative disorders, are associated with metabolic defects [28], [29]. *Drosophila* has been used successfully to model neurodegenerative disorders by expressing pathogenic forms of disease-causing genes in the fly's nervous system [30]. Interestingly, when we expressed Aβ42ArcM, a pathogenic form of the amyloid protein shown to induce neurodegeneration [21], in the central clock neurons using *Pdf-Gal4*, fat body triglycerides were elevated similarly to when CLKΔ and *Clk*RNAi were expressed in the same neurons (Fig. 3B). No circadian behavioral phenotype was observed in the Aβ42ArcM-expressing animals, suggesting that some function of the ventral lateral neurons, but not necessarily the clock in them, is important for regulating fat body triglycerides (Table 1).

**Figure 4:**
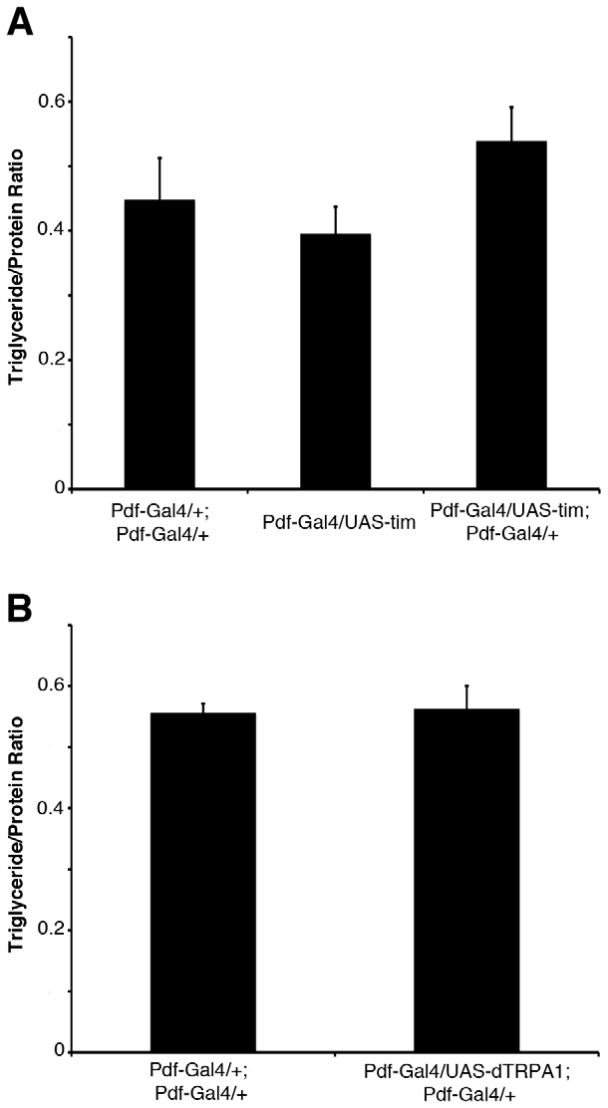
Expression of *tim* and dTRPA1 in the PDF neurons has no effect on fat body triglycerides. (A) Triglyceride/protein ratios of fat bodies dissected from 4–7 day old *Pdf-Gal4/UAS-tim* and *Pdf-Gal4/UAS-tim*; *Pdf-Gal4/+* female flies and compared to *Pdf-Gal4/+*; *Pdf-Gal4/+* controls. Animals from each genotype were dissected at ZT8 and 20 and the values from the two time points were averaged. (B) Triglyceride/protein ratios of fat bodies dissected from 7–10 day old *Pdf-Gal4/UAS-dTRPA1*; *Pdf-Gal4/+* female flies at ZT 0–2 and compared to *Pdf-Gal4/+*; *Pdf-Gal4/+* controls at ZT 0–2. Each experiment was performed at least three times and values represent mean ± SEM. The data in (A) were obtained from 120 animals and the data in (B) from 45 animals for each genotype.

To further address the role of the clock in PDF neurons in the control of fat body lipid storage, we sought to measure triglycerides in response to other genetic manipulations that alter activity rhythms. Our lab showed previously that preventing *tim* cycling by over-expressing it in all clock neurons abolishes behavioral rhythms [22]. Over-expression of *tim* in PDF neurons alone does not eliminate rhythms altogether, but leads to an increased incidence of arrhythmia (Table 1); however, no change in fat body triglycerides was observed (Fig. 4A). Additionally, no lipid phenotype was observed when the heat-sensitive Na^+^ channel, dTRPA1, was used to increase excitability of the PDF neurons (Fig. 4B), even though this manipulation resulted in an arrhythmic behavioral rhythm phenotype, similar to that seen when the bacterial Na^+^ channel, NaChBac was expressed in the same neurons (Table 1, [31]). Together these data suggest that altering the function of the PDF neurons leads to increased fat body triglycerides, but the circadian function of these neurons may not be important for this purpose.

### Increased feeding does not account for the increased lipid phenotype observed when PDF neuron function is altered

**Figure 5:**
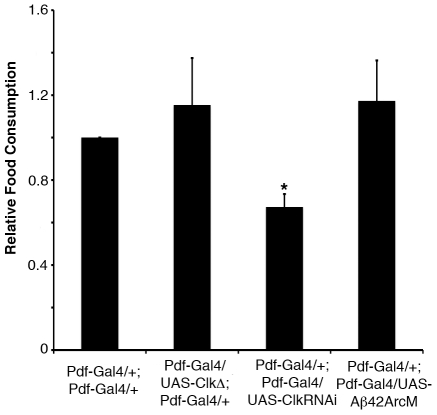
The effect of manipulating the PDF neurons on food consumption. Total food consumption over a 24-hour period of 4–7 day old *Pdf-Gal4/UAS-ClkΔ*; *Pdf-Gal4/+*, *Pdf-Gal4/+*; *Pdf-Gal4/UAS-Clk*RNAi, and *Pdf-Gal4/+*; *Pdf-Gal4/UAS-*Aβ42ArcM female flies and normalized to *Pdf-Gal4/+*; *Pdf-Gal4/+*controls. Each experiment was performed three times with at least 40 animals for each genotype. Values represent mean ± SEM. * p<0.05 assessed by Student's t-test compared to *Pdf-Gal4/+*; *Pdf-Gal4/+* controls.

In addition to the storage of glycogen and lipids, feeding behavior is controlled by the circadian system [7], [15]. While recent evidence points to the fat body clock as a major circadian regulator of feeding in *Drosophila*, the contribution of the central clock to controlling feeding has not been addressed. Therefore, it is possible that the increased fat phenotype observed above is a result of increased feeding. To test this hypothesis, we measured food consumption over a 24-hr period in animals expressing CLKΔ, *Clk*RNAi, and Aβ42ArcM in the PDF neurons. The total food intake of these animals was not higher than that of controls; in fact, the *Clk*RNAi flies had less food consumption than controls (Fig. 5), indicating that increased feeding does not account for the high lipids observed in animals with altered clock neurons.

## Discussion

In this study, we established a role for the PDF^+^ circadian neurons in controlling lipid storage non-cell-autonomously in the fat body. The effects on lipid levels reported here are consistent with our previously published data on the control of glycogen levels by neuronal clocks [15]. In addition, we have identified specific neurons – those that house the central clock – that account for at least some of the neuronal effects on triglycerides. Given this apparent control of lipid metabolism by the circadian neurons, one might expect that fat body triglycerides would cycle throughout the circadian day, but data presented here show this is not the case. One possibility is that triglyceride cycling occurs, but cannot be detected with current methodology. Another possibility is that regulation of fat body triglycerides by multiple clocks prevents cyclic control that may be exerted by one clock alone. This would allow for maintenance of constant levels, which may be more beneficial for the organism. Finally, as noted below, the current study favors a non-circadian effect of the LNvs on fat body triglycerides, which would also explain the lack of cycling.

The dominant-negative CLK protein data, and even the RNAi knockdown data, implicate the circadian clock in controlling lipid storage. This would be consistent with previous reports on the control of lipid metabolism by the circadian clock in mammals [6], [7]. However, we elicit a similar triglyceride increase when the Alzheimer's Disease-causing protein Aβ42ArcM is expressed in the PDF neurons, and this manipulation has no effect on the central clock as evidenced by normal activity behavior (Table 1). Conversely, expression of *tim* and dTRPA1 in the clock neurons affects circadian function by altering activity rhythms (Table 1), but has no effect on fat body triglycerides (Fig. 4). While it is possible that *tim* over-expression in PDF neurons alone is not strong enough to affect triglycerides (the effect on behavioral rhythms in this case is weaker than that of *tim* over-expression by the *tim*-Gal4 driver [22]), and that dTRPA1 affects specific outputs from the neurons rather than the clock itself, a more likely scenario is that the circadian clock is not involved in eliciting the triglyceride phenotype described in this study. The effect of *Clk* shown here would then reflect a non-circadian function of this gene. Additionally, the major circadian output signal of LNvs, PDF, does not appear to be involved in the control of fat body lipids either as *Pdf^01^* mutants have normal triglycerides (Fig. 2E). This is consistent with a recent study demonstrating that a PDF-independent factor mediates part of the complex rhythm observed when the electrical activity of the LNvs is altered [32].

One outstanding question raised by this study is how the central clock neurons communicate with the fat body to regulate lipid metabolism. One possibility is that the circadian neurons regulate overall feeding behavior, which would then secondarily affect lipid storage. However, this mechanism seems unlikely, as expressing CLKΔ, *Clk*RNAi or Aβ42ArcM in the PDF neurons increases fat body triglycerides but doesn't increase the amount of food consumed over a 24-hr period (Figs. 2, 3, 5). Another potential mechanism is that the fat body receives signals from the brain to control lipid metabolism through direct neuronal connections. In mammals, the sympathetic nervous system innervates white adipose tissue and is thought to be a major initiating stimulus for lipid mobilization [33], [34]. A similar circuit might be present in *Drosophila*, although whether the fat body is innervated by the fly's nervous system is still unknown. A third possibility is that the PDF neurons or another neuronal population that receives signals from the PDF neurons produces a secreted factor that travels through the hemolymph and acts on the fat body to regulate lipid levels. While it seems that PDF is dispensable for controlling lipid levels since *Pdf^01^* mutants have normal triglycerides (Fig. 2E), other peptides are expressed in the fly brain such as the insulin-like peptides (DILPs), the feeding peptide neuropeptide F (NPF), and a number of novel peptides whose functions are still unclear [35], [36], [37]. Recent studies showed that the PDF neurons express NPF, which based upon the role of NPF in regulating feeding and metabolism, could be a potential mediator of the lipid storage phenotype described here [36], [38], [39]. In any case, this study implicates the central clock neurons in controlling fat body triglycerides and demonstrates the utility of the *Drosophila* system to increase our understanding of the mechanisms whereby specific populations of neurons regulate lipid metabolism.

## References

[1] Stein CJ, Colditz GA (2004). The epidemic of obesity.. J Clin Endocrinol Metab.

[2] Kohlwein SD (2010). Triacylglycerol homeostasis: insights from yeast.. J Biol Chem.

[3] Benarroch EE (2010). Neural control of feeding behavior: Overview and clinical correlations.. Neurology.

[4] Nogueiras R, Lopez M, Dieguez C (2010). Regulation of lipid metabolism by energy availability: a role for the central nervous system.. Obes Rev.

[5] Saeb-Parsy K, Lombardelli S, Khan FZ, McDowall K, Au-Yong IT (2000). Neural connections of hypothalamic neuroendocrine nuclei in the rat.. J Neuroendocrinol.

[6] Rudic RD, McNamara P, Curtis AM, Boston RC, Panda S (2004). BMAL1 and CLOCK, two essential components of the circadian clock, are involved in glucose homeostasis.. PLoS Biol.

[7] Turek FW, Joshu C, Kohsaka A, Lin E, Ivanova G (2005). Obesity and metabolic syndrome in circadian Clock mutant mice.. Science.

[8] Damiola F, Le Minh N, Preitner N, Kornmann B, Fleury-Olela F (2000). Restricted feeding uncouples circadian oscillators in peripheral tissues from the central pacemaker in the suprachiasmatic nucleus.. Genes Dev.

[9] Le Martelot G, Claudel T, Gatfield D, Schaad O, Kornmann B (2009). REV-ERBalpha participates in circadian SREBP signaling and bile acid homeostasis.. PLoS Biol.

[10] Vollmers C, Gill S, DiTacchio L, Pulivarthy SR, Le HD (2009). Time of feeding and the intrinsic circadian clock drive rhythms in hepatic gene expression.. Proc Natl Acad Sci U S A.

[11] Baker KD, Thummel CS (2007). Diabetic larvae and obese flies-emerging studies of metabolism in Drosophila.. Cell Metab.

[12] Allada R, Chung BY (2010). Circadian organization of behavior and physiology in Drosophila.. Annu Rev Physiol.

[13] Chatterjee A, Tanoue S, Houl JH, Hardin PE (2010). Regulation of gustatory physiology and appetitive behavior by the Drosophila circadian clock.. Curr Biol.

[14] Tanoue S, Krishnan P, Krishnan B, Dryer SE, Hardin PE (2004). Circadian clocks in antennal neurons are necessary and sufficient for olfaction rhythms in Drosophila.. Curr Biol.

[15] Xu K, Zheng X, Sehgal A (2008). Regulation of feeding and metabolism by neuronal and peripheral clocks in Drosophila.. Cell Metab.

[16] Allada R, White NE, So WV, Hall JC, Rosbash M (1998). A mutant Drosophila homolog of mammalian Clock disrupts circadian rhythms and transcription of period and timeless.. Cell.

[17] Rutila JE, Suri V, Le M, So WV, Rosbash M (1998). CYCLE is a second bHLH-PAS clock protein essential for circadian rhythmicity and transcription of Drosophila period and timeless.. Cell.

[18] Myers MP, Wager-Smith K, Wesley CS, Young MW, Sehgal A (1995). Positional cloning and sequence analysis of the Drosophila clock gene, timeless.. Science.

[19] Konopka RJ, Benzer S (1971). Clock mutants of Drosophila melanogaster.. Proc Natl Acad Sci U S A.

[20] Renn SC, Park JH, Rosbash M, Hall JC, Taghert PH (1999). A pdf neuropeptide gene mutation and ablation of PDF neurons each cause severe abnormalities of behavioral circadian rhythms in Drosophila.. Cell.

[21] Iijima K, Chiang HC, Hearn SA, Hakker I, Gatt A (2008). Abeta42 mutants with different aggregation profiles induce distinct pathologies in Drosophila.. PLoS One.

[22] Yang Z, Sehgal A (2001). Role of molecular oscillations in generating behavioral rhythms in Drosophila.. Neuron.

[23] Hamada FN, Rosenzweig M, Kang K, Pulver SR, Ghezzi A (2008). An internal thermal sensor controlling temperature preference in Drosophila.. Nature.

[24] DiAngelo JR, Birnbaum MJ (2009). Regulation of fat cell mass by insulin in Drosophila melanogaster.. Mol Cell Biol.

[25] Hoshizaki DK, Gilbert LI, Iatrou K, Gill SS (2005). Fat-Cell Development.. Comprehensive Molecular Insect Science.

[26] Park JH, Helfrich-Forster C, Lee G, Liu L, Rosbash M (2000). Differential regulation of circadian pacemaker output by separate clock genes in Drosophila.. Proc Natl Acad Sci U S A.

[27] Koh K, Evans JM, Hendricks JC, Sehgal A (2006). A Drosophila model for age-associated changes in sleep∶wake cycles.. Proc Natl Acad Sci U S A.

[28] Bjorkhem I, Leoni V, Meaney S (2010). Genetic connections between neurological disorders and cholesterol metabolism.. J Lipid Res.

[29] de la Monte SM, Longato L, Tong M, Wands JR (2009). Insulin resistance and neurodegeneration: roles of obesity, type 2 diabetes mellitus and non-alcoholic steatohepatitis.. Curr Opin Investig Drugs.

[30] Lessing D, Bonini NM (2009). Maintaining the brain: insight into human neurodegeneration from Drosophila melanogaster mutants.. Nat Rev Genet.

[31] Nitabach MN, Wu Y, Sheeba V, Lemon WC, Strumbos J (2006). Electrical hyperexcitation of lateral ventral pacemaker neurons desynchronizes downstream circadian oscillators in the fly circadian circuit and induces multiple behavioral periods.. J Neurosci.

[32] Sheeba V, Sharma VK, Gu H, Chou YT, O'Dowd DK (2008). Pigment dispersing factor-dependent and -independent circadian locomotor behavioral rhythms.. J Neurosci.

[33] Bartness TJ, Bamshad M (1998). Innervation of mammalian white adipose tissue: implications for the regulation of total body fat.. Am J Physiol.

[34] Bartness TJ, Song CK (2007). Thematic review series: adipocyte biology. Sympathetic and sensory innervation of white adipose tissue.. J Lipid Res.

[35] Rulifson EJ, Kim SK, Nusse R (2002). Ablation of insulin-producing neurons in flies: growth and diabetic phenotypes.. Science.

[36] Wu Q, Wen T, Lee G, Park JH, Cai HN (2003). Developmental control of foraging and social behavior by the Drosophila neuropeptide Y-like system.. Neuron.

[37] Verleyen P, Baggerman G, Wiehart U, Schoeters E, Van Lommel A (2004). Expression of a novel neuropeptide, NVGTLARDFQLPIPNamide, in the larval and adult brain of Drosophila melanogaster.. J Neurochem.

[38] Kula-Eversole E, Nagoshi E, Shang Y, Rodriguez J, Allada R Surprising gene expression patterns within and between PDF-containing circadian neurons in Drosophila.. Proc Natl Acad Sci U S A.

[39] Lee G, Bahn JH, Park JH (2006). Sex- and clock-controlled expression of the neuropeptide F gene in Drosophila.. Proc Natl Acad Sci U S A.

